# A Reexamination of the PPAR-α Activation Mode of Action as a Basis for Assessing Human Cancer Risks of Environmental Contaminants

**DOI:** 10.1289/ehp.0900758

**Published:** 2009-05-15

**Authors:** Kathryn Z. Guyton, Weihsueh A. Chiu, Thomas F. Bateson, Jennifer Jinot, Cheryl Siegel Scott, Rebecca C. Brown, Jane C. Caldwell

**Affiliations:** National Center for Environmental Assessment, Office of Research and Development, U.S. Environmental Protection Agency, Washington, DC, USA

**Keywords:** carcinogenesis, mode of action, peroxisome proliferators, risk assessment

## Abstract

**Background:**

Diverse environmental contaminants, including the plasticizer di(2-ethylhexyl)phthalate (DEHP), are hepatocarcinogenic peroxisome proliferators in rodents. Peroxisome proliferator–activated receptor-α (PPAR-α) activation and its sequelae have been proposed to constitute a mode of action (MOA) for hepatocarcinogenesis by such agents as a sole causative factor. Further, based on a hypothesized lower sensitivity of humans to this MOA, prior reviews have concluded that rodent hepatocarcinogenesis by PPAR-α agonists is irrelevant to human carcinogenic risk.

**Data synthesis:**

Herein, we review recent studies that experimentally challenge the PPAR-α activation MOA hypothesis, providing evidence that DEHP is hepatocarcinogenic in PPAR-α–null mice and that the MOA but not hepatocarcinogenesis is evoked by PPAR-α activation in a transgenic mouse model. We further examine whether relative potency for PPAR-α activation or other steps in the MOA correlates with tumorigenic potency. In addition, for most PPAR-α agonists of environmental concern, available data are insufficient to characterize relative human sensitivity to this rodent MOA or to induction of hepatocarcinogenesis.

**Conclusions:**

Our review and analyses raise questions about the hypothesized PPAR-α activation MOA as a sole explanation for rodent hepatocarcinogenesis by PPAR-α agonists and therefore its utility as a primary basis for assessing human carcinogenic risk from the diverse compounds that activate PPAR-α. These findings have broad implications for how MOA hypotheses are developed, tested, and applied in human health risk assessment. We discuss alternatives to the current approaches to these key aspects of mechanistic data evaluation.

Data on a chemical’s mechanisms of action can be critical to decisions in human health risk assessment, especially regarding potential differences in sensitivity to adverse effects across and within species. U.S. Environmental Protection Agency (EPA) guidelines for carcinogen risk assessment (U.S. EPA 2005) particularly emphasize the use of mechanistic data to inform the evaluation of the mode of action (MOA) of a chemical. These guidelines define MOA as “a sequence of key events and processes, starting with interaction of an agent with a cell, proceeding through operational and anatomical changes and resulting in cancer formation.” A “key event” is an empirically observable precursor step that is itself a necessary element of the MOA or is a biologically based marker for such an element. Mode is distinguished from mechanism, with the latter implying the complete series of events that lead from exposure to disease. It is recognized that the precise carcinogenic mechanism(s) is unknown for essentially all chemicals, and the MOA concept attempts to address this knowledge deficit by focusing on key steps required (i.e., necessary) for the carcinogenic process.

One of the most challenging determinations in evaluating mechanistic data is whether an identified MOA is the cause of cancer (or another adverse health outcome). As recently reviewed (Guyton et al. 2008), the U.S. EPA and other MOA approaches have adopted the Hill (1965) considerations to evaluate hypothesized MOAs. However, the Hill considerations were not developed for this purpose. They were instead designed to assist epidemiologists in making inductive inferences about the causal relationship of observed associations or, in the language of chemical carcinogenesis, as an aid in addressing the question, “Does a chemical exposure cause cancer?” Causal inference is often used in epidemiology because direct experimentation is generally not feasible; in contrast, MOA questions—for example, “How does a chemical cause cancer?”—are almost entirely addressed through empirical tests of the pertinent hypotheses in laboratory studies. Although some of the Hill considerations (e.g., temporality and dose–response relationships) may be helpful in ruling out implausible MOAs, their general utility for elucidating if biological effects that may be similarly related to exposure (e.g., cytotoxicity) are actual “key events” in the causal pathway(s) to cancer deserves scrutiny. It is unclear whether they can distinguish effects that are merely associated with cancer (by virtue of a common causal connection with the exposure) from effects that are causally linked as part of an MOA for carcinogenesis. Moreover, even when particular effects are key events in a causal pathway to cancer, the Hill considerations cannot rule out the causal contribution of other effects or pathways. In order to address these types of inquiry, direct experimentation is needed, and MOA hypotheses should be empirically challenged using experimental studies, in accordance with the scientific method.

Another difficult aspect of mechanistic data evaluation is the assessment of likely human sensitivity to an identified laboratory animal MOA. The U.S. EPA (2005) guidelines address this issue separately during the hazard characterization and dose–response components of risk assessment, as well as in the identification of susceptible populations. This approach avoids conflating qualitative and quantitative issues during hazard characterization, and thereby prevents human relevance from being ruled out based on differences in sensitivity across species. Instead, information suggesting quantitative differences is “flagged for consideration in the dose–response assessment” and quantitative adjustments may also be made based on identified susceptibilities (e.g., life stage). However, a limitation of this approach and of MOA approaches in general is the tacit assumption of site concordance, which departs from the basic tenet of U.S. EPA cancer risk assessment that site concordance across species is not required in hazard evaluation, a tenet consistent with observations of site disconcordance across countless rodent bioassays.

We explore these aspects of MOA evaluation in consideration of peroxisome proliferator–activated receptor-α (PPAR-α) activation as an MOA for rodent liver tumor carcinogenesis. This MOA has been invoked to support the conclusion that environmental contaminants [e.g., the plasticizer di(2-ethylhexyl) phthalate (DEHP)] would lack carcinogenic risk in humans. For instance, the International Agency for Research on Cancer (IARC) downgraded the classification of DEHP from “possibly carcinogenic to humans” to “not classifiable” based on an expert panel’s acceptance of the hypothesis that DEHP induces liver tumors in rodents by a mechanism dependent on PPAR-α activation that is not relevant to humans (IARC 2000). The use of evidence of the PPAR-α activation MOA to dismiss the human relevance of effects observed in laboratory animals has been questioned (e.g., Caldwell et al. 2008; Melnick 2001) based on the lack of experimental studies empirically challenging the MOA hypothesis.

Two recent studies have provided evidence that DEHP induces liver tumors in PPAR-α–null mice (Ito et al. 2007a) and that hepatocyte PPAR-α activation (and its sequelae) in a transgenic mouse model is insufficient to evoke hepatocarcinogenesis (Yang et al. 2007). These data provide motivation for the present reexamination of the evidential support for the proposed PPAR-α activation MOA hypothesis as a basis for assessing human health risks of DEHP and other environmental contaminants that activate PPAR-α. Although we focus on the human health risk assessment implications of recent data rather than comprehensively reviewing the topic area, our analyses also encompass some prior studies reviewed more extensively elsewhere (Ashby et al. 1994; Klaunig et al. 2003; Melnick 2001). Particularly, we examine the extent to which the quantitative relationship between the proposed key events and liver tumorigenesis in rodents, as well as the relative human sensitivity to the hypothesized rodent MOA, have been characterized. In addition, we consider whether the available epidemiologic evidence on fibrates affords a test of the supposition that laboratory animal data about PPAR-α agonists indicate the absence of human health risks. We conclude with a discussion of alternative approaches to analyses of mechanistic information for chemicals of environmental concern that could be considered to address some of the challenges elaborated herein.

## Is PPAR-α Activation Essential for DEHP Liver Carcinogenesis?

**Figure 1:**
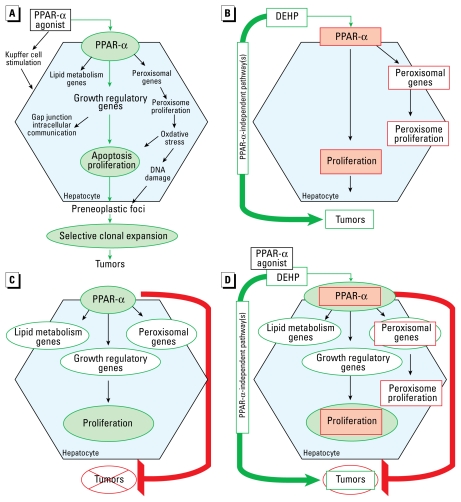
(*A*) Hypothesized PPAR-α activation MOA as posited by Klaunig et al. (2003), with proposed “causal” events identified in green. (*B*) Is PPAR-α activation essential for DEHP carcinogenesis? Red outlines represent key events in the hypothesized PPAR-α activation MOA that were not induced by DEHP in PPAR-α–null mice despite the occurrence of tumors (Ito et al. 2007a; Ward et al. 1998). Proposed causal events are shaded pink. These key events are therefore not necessary for tumors, suggesting PPAR-α–independent pathways for DEHP hepatocarcinogenesis. (*C*) Is PPAR-α activation alone sufficient for carcinogenesis? In the Yang et al. (2007) LAP-VP16PPAR-α transgenic model of constitutive PPAR-α activation in hepatocytes, the key events in the hypothesized PPAR-α activation MOA (green outlines), but not tumors, are induced at 11 months. Proposed causal events in the MOA are shaded light green. Wy-14,643 exposure in wild-type mice induces tumors at 11 months with comparable levels of hepatocyte proliferation and other proposed key events. This raises questions about whether PPAR-α activation and hepatocyte proliferation can alone cause tumors, and suggests that the sequence of key events in the hypothesized MOA is not solely sufficient to evoke carcinogenesis. (*D*) Revisiting the PPAR-α activation MOA. DEHP is hepatocarcinogenic in PPAR-α–null mice in which the red-outlined key events are absent (Ito et al. 2007a; Ward et al. 1998), whereas, the green-outlined key events, but not tumors, are induced at 11 months in the LAP-VP16PPAR-α transgenic model (Yang et al. 2007). Proposed causal events are shaded light green. Taken together, these findings support the view that the hypothesized PPAR-α activation MOA is neither necessary nor sufficient for hepatocarcinogenesis as a sole causative factor.

Figure 1A shows a widely cited version of the hypothesized MOA for hepatocarcinogenesis induced by PPAR-α agonists, posited by Klaunig et al. (2003), in which three key causal events were proposed: activation of the receptor, perturbation of hepatocellular apoptosis and proliferation, and selective clonal expansion. Klaunig et al. (2003) concluded that PPAR-α activation represents a key event in the liver tumor MOA for DEHP. The evidential support included the *in vitro* demonstration of receptor activation by its monoester metabolite [mono-2-ethylhexyl phthalate (MEHP)] (Issemann and Green 1990; Maloney and Waxman 1999). In addition, peroxisome proliferation (or increased peroxisomal enzyme activity), an associative event in the MOA, was induced by tumorigenic doses of DEHP in the liver of mice and rats or by MEHP in rat hepatocytes (David et al. 1999; Gray et al. 1982, 1983; Hasmall et al. 1999; Mitchell et al. 1984, 1985; Reddy et al. 1986). Comparable to the results found for prototypical agonist [4-chloro-6-(2,3-xylidino)-2-pyrimidyl-thio]acetic acid (Wy-14,643), DEHP failed to induce peroxisomal enzymes and peroxisome proliferation in PPAR-α–null mice with 24 weeks of exposure (Ward et al. 1998). This finding supported a causal role for receptor activation in peroxisome proliferation, but a study of the subsequent development of tumors by DEHP in PPAR-α–null mice had not been conducted. However, Peters et al. (1997) had previously reported the absence of tumors in nine PPAR-α–null mice exposed to Wy-14,643 at 11 months, whereas each of the six similarly exposed wild-type mice had multiple hepatocellular neoplasms. Based on this 11-month tumor bioassay of Wy-14,643 (Peters et al. 1997) and the short-term DEHP study (Ward et al. 1998) in PPAR-α–null mice, it was assumed that “a long-term study of DEHP using PPAR-α–null mice would provide the same results” (Klaunig et al. 2003). This assumption in turn supported the conclusion that “all the effects observed are due only to the activation of this receptor and the downstream events resulting from this activation and that no other modes of action are operant” (Klaunig et al. 2003).

**Figure 2:**
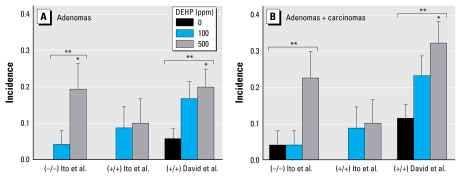
Incidences of hepatocellular adenomas (*A*) and hepatocellular adenomas and carcinomas (*B*) in mice exposed to DEHP. Ito et al. (2007a) exposed PPAR-α–null (−/−) and wild-type (+/+) Sv129 mice for 22 months; David et al. (1999) exposed B6C3F1 wild-type (+/+) mice for up to 104 weeks. Data are presented as incidence ± SD assuming a binomial distribution for each group. All pairwise cross-study comparisons between like dose groups [e.g., Ito et al. 2007a (−/−) 500 ppm vs. David et al. (+/+) 500 ppm] were not significant by Fisher exact test. Because David et al. reported only adenomas and carcinomas, we excluded from analyses the cholangiocellular carcinoma reported by Ito et al. in DEHP-exposed PPAR-α–null mice. *Significantly different from controls of the same genotype in the same study (Fisher exact test, *p* < 0.05). **Significant trend with dose in the study (Cochran Armitage test, *p* < 0.05).

Recent studies have experimentally challenged both the assumption of a lack of carcinogenicity in a long-term DEHP bioassay in PPAR-α–null mice and the hypothesis that PPAR-α activation is the sole operant MOA for hepatocarcinogenesis. In a 2-year bioassay, Ito et al. (2007a) found that DEHP (100 or 500 ppm) induces liver tumors in PPAR-α–null mice and they also reported a significant trend for the observed increase in total liver tumors with DEHP in PPAR-α–null male mice with Sv129 genetic background generated as described in Lee et al. (1995). For the present report, we performed additional statistical analyses to compare the Ito et al. (2007a) results with those of a prior DEHP bioassay in B6C3F1 wild-type mice (David et al. 1999). A pairwise analysis showed that DEHP (500 ppm) significantly increased adenomas in PPAR-α–null mice but not in companion wild-type mice compared with their respective controls (Figure 2). In the David et al. study of B6C3F1 mice, DEHP (500 ppm) also significantly increased adenomas and adenomas plus carcinomas (Figure 2). Moreover, we found a significant dose–response trend for adenomas and for adenomas plus carcinomas in both the Ito et al. PPAR-α–null mice and the David et al. B6C3F1 mice after exposure to DEHP (Figure 2). Additionally, we found no statistically significant differences between groups at the same dose, including controls, consistent with mouse strain and PPAR-α genotype having no influence on carcinogenicity under the study conditions.

The observed lack of difference in reported control incidences across groups lends support to our approach of basing comparative analyses on concurrent controls. Historical data on spontaneous liver tumor incidences in PPAR-α–null mice are limited; Ito et al. (2007a) is the largest published 2-year bioassay in PPAR-α–null mice, reporting findings for 24 of 25 surviving unexposed animals at 23 months of age. A different laboratory that had established a distinct breeding colony reported mouse liver tumor incidences in 12 PPAR-α–null Sv129/C57BL/6 mice approximately 2 years of age (Howroyd et al. 2004). Adenomas and carcinomas were reported in 6 of 12 and 2 of 12 PPAR-α–null mice, respectively, compared with adenomas in 5 of 22 wild-type animals. As Howroyd et al. noted, “The relatively small number of animals available made it difficult to draw robust conclusions concerning enhancement of spontaneous findings in PPARα-null mice.” In addition, the low survival of Howroyd et al.’s PPAR-α–null mice relative to those of Ito et al. (2007a) limit statistical comparisons based on this data set.

In sum, the Ito et al. (2007a) study is consistent with the view that DEHP carcinogenesis can occur independently of PPAR-α activation. As noted in a report by the National Research Council (2008a), this finding “calls into question” the IARC conclusions regarding the carcinogenic risks of DEHP (IARC 2000). Indeed, as summarized in Figure 1B, PPAR-α activation and the subsequent key events in the hypothesized MOA do not appear to represent the sole cause of DEHP liver tumorigenesis. Although new hypotheses are being generated based on more detailed comparisons between wild-type and PPAR-α–null mice (Ito et al. 2007a; Takashima et al. 2008), the mechanisms by which DEHP induces hepatocarcinogenesis remain unknown.

## Do PPAR-α Activation and Its Sequelae in Hepatocytes Cause Cancer?

A second recent study raises further questions regarding whether PPAR-α activation in hepatocytes is causally linked to hepatocarcinogenesis as a sole operant MOA (Yang et al. 2007). The experimental approach entailed fusing the mouse PPAR-α to the potent viral transcriptional activator VP16 under control of the liver-enriched activator protein (LAP) promoter, resulting in targeted constitutive expression of activated PPAR-α in hepatocytes. In LAP-VP16PPAR-α transgenic mice, many of the same hepatic responses (in type and magnitude) were seen as with PPAR-α ligand treatment of companion wild-type Sv129 mice. For instance, DNA synthesis was increased in LAP-VP16PPAR-α transgenic mice; the effect was persistent and still evident at 11 months of age. In addition, increases were reported in markers of peroxisome proliferation [including increases in expression of peroxisomal membrane protein 70, acyl coenzyme A (CoA) oxidase and cytochrome P4A (CYP4A) family genes, and enhanced cyanide-insensitive palmitoyl CoA oxidation]. Other effects included an increase in cell-cycle genes (cyclin D1 and cyclin-dependent kinase-1 and -4) and a decrease in serum triglycerides and free fatty acids. Together, the results from this transgenic model are consistent with the view that PPAR-α activation and its sequelae are alone sufficient to induce increased hepatocyte DNA synthesis (a key event in the hypothesized MOA) as well as peroxisome proliferation.

However, constitutive PPAR-α activation in hepatocytes in the LAP-VP16PPAR-α transgenic mouse model was not sufficient to induce several important hepatic responses stimulated by PPAR-α ligand treatment of wild-type mice. Notably, Yang et al. (2007) found no preneoplastic hepatic lesions or hepatocellular neoplasia in “> 20 LAP-VP16PPAR-α mice at the age of < 1 year.” In sharp contrast, wild-type mice exposed to the PPAR-α agonist Wy-14,643 for 11 months developed grossly visible lesions consistent with previous reports of its hepatocarcinogenicity (e.g., Peters et al. 1997). Interestingly, nonparenchymal cell proliferation was seen with Wy-14,643 exposure of wild-type mice but was absent in the LAP-VP16PPAR-α transgenic mice. In addition, although liver weight was increased in LAP-VP16PPAR-α transgenic mice, the extent of hepatomegaly was reduced compared with Wy-14,643–exposed wild-type mice, and hepatocellular hypertrophy was absent.

Thus, the Yang et al. (2007) study provides evidence that, by itself, PPAR-α activation (and its sequelae) is not sufficient to induce hepatocarcinogenesis. These data are therefore inconsistent with the hypothesis that effects mediated through PPAR-α activation constitute a complete MOA for carcinogenesis (Figure 1C). Notably, key events in the proposed MOA such as the robust and sustained elevation in hepatocyte proliferation (evidenced by enhanced DNA synthesis), accompanied by enzyme changes commonly associated with peroxisome proliferation, did not evoke hepatocarcinogenesis. In fact, a comparable extent of sustained increases in hepatocyte DNA synthesis was seen with constitutive PPAR-α activation in the LAP-VP16PPAR-α transgenic mouse model and Wy-14,643 exposure in wild-type mice, but only the latter developed liver tumors under comparable experimental paradigms.

These observations indicate that events besides the prolonged elevation of proliferation (evidenced by an increase in DNA synthesis) are necessary in the carcinogenic MOA for Wy-14,643, a compound frequently used as the model for PPAR-α agonism. Moreover, in contrast to Wy-14,643, DEHP only transiently increases DNA synthesis and at higher doses than required for carcinogenesis (Conway et al. 1989; David et al. 1999; Marsman et al. 1988) (Table 1). Only short-term increases in rat liver DNA synthesis are seen with many other PPAR-α agonists, including clofibrate [ethyl 2-(4-chlorophenoxy)-2-methylpropanoate] (Tanaka et al. 1992), clofibric acid [2-(*p*-chlorophenoxy)-2-methylpropionic acid] (Barrass et al. 1993; Marsman et al. 1992), nafenopin (2-methyl-2[*p*-(1,2,3,4-tetrahydro-1-naphthyl)phenoxy]-propionic acid) (Eacho et al. 1991; Lake et al. 1993), ciprofibrate (2-[4-(2,2-dichlorocyclopropyl)phenoxy]-2-methylpropanoic acid) (Yeldandi et al. 1989), and trichloroacetic acid (TCA) (Walgren et al. 2005). A causal mechanistic link between transient increases in DNA synthesis and rodent hepatocarcinogenesis remains to be established (Melnick et al. 1993).

### Are key or associative events in the PPAR-α activation MOA quantitatively predictive of hepatocarcinogenicity?

Another question to consider is whether potency for PPAR-α activation or its attendant sequelae is quantitatively associated with carcinogenic activity or potency. If so, differences in sensitivity for carcinogenesis (such as may occur across species) could be predicted using quantitative information about the key events alone. However, there are limitations in the dose–response data available for analyses of quantitative relationships between potencies for precursor events in the proposed PPAR-α activation MOA and for liver tumor induction. Most tumor data, including for the best-characterized PPAR-α agonists, are for exposure concentrations inducing well above 50% carcinogenic potency (TD_50_; the daily dose inducing tumors in half of the mice that would otherwise have remained tumor-free) with less-than-lifetime administration. Precursor events have typically been studied at a single dose, often eliciting a near maximal response, thus precluding benchmark-based comparisons across studies. This is especially true for Wy-14,643, which has been administered most often at only one exposure concentration (1,000 ppm) that elicits a 100% tumor incidence after 1 year or less (Peters et al. 1997) and that also appears to be necrogenic (Woods et al. 2007). On the other hand, hypothesized precursor events such as hepatomegaly, peroxisome proliferation, and increased DNA synthesis appear to have reached their maximal responses at 50 ppm Wy-14,643, with some statistically significant responses at doses as low as 5 ppm (Marsman et al. 1992; Wada et al. 1992). Potencies across compounds have rarely been compared in a single study using the same experimental paradigm. These deficits in the database notwithstanding, we provide below an assessment of the quantitative predictive power of the potency for four proposed data elements for establishing the hypothesized MOA for hepatocarcinogenesis: PPAR-α activation in mice, and hepatomegaly, DNA synthesis, and increased peroxisome proliferation in rats.

### PPAR-α activation in mice

Table 2 presents data for four peroxisome proliferators in order of decreasing potency for inducing mouse liver tumors. We selected these compounds because of their importance to environmental human health risk assessments and because data to derive receptor activation potency indicators were available from a single study (Maloney and Waxman 1999). The trans-activation potencies of MEHP, Wy-14,643, dichloroacetic acid (DCA), and TCA for the mouse PPAR-α were monitored using a luciferase reporter gene containing multiple PPAR response elements derived from the rat hydratase/dehydrogenase promoter in transiently transfected COS-1 monkey kidney cells. The derived potency indicators were compared with the TD_50_ from the Carcinogenic Potency Database (CPDB) of Gold et al. (2005). Note that for Wy-14,643, the dose listed yielded a maximal response and thus represents an upper limit to the TD_50_ (indicated by “<“). Two estimates of PPAR-α transactivation potency are given, the first based on the effective concentration yielding 50% of the maximal response (EC_50_) and the second based on the effective concentration required for a 2-fold increase in activity (EC_2-fold_) (Maloney and Waxman 1999). Orally administered DEHP undergoes presystemic hydrolysis catalyzed by lipase to MEHP in the gut, with mice exhibiting higher lipase activities in the small intestine compared with rats and marmosets (Ito et al. 2005; Kessler et al. 2004; Pollack et al. 1985). Therefore, because the mouse liver is likely exposed predominantly to MEHP rather than DEHP and because unmetabolized DEHP does not exhibit PPAR-α activity, the transactivation activity of its metabolite MEHP is given but compared with the hepatocarcinogenic potency indicator for DEHP. No data on the potency for transactivation of rat PPAR-α by chemicals in the CPDB were located to enable a similar comparison in rats.

These data clearly show a lack of correlation between the potencies for *in vitro* PPAR-α transactivation and *in vivo* tumorigenesis across different PPAR-α agonists. Especially notable is that MEHP exhibited orders of magnitude more potency for transactivating mouse PPAR-α than does DCA, but DEHP was 6-fold less potent as a mouse hepatocarcinogen. TCA was more similar in potency to DCA for both outcomes; that is, it was also dramatically less active at transactivating PPAR-α than DEHP despite exhibiting comparable hepatocarcinogenic potency. Wy-14,643 and MEHP activate PPAR-α at comparable concentrations when directly compared in the transactivation assay, but the carcinogenic potency of Wy-14,643 was estimated to be at least 70-fold higher than that of DEHP. This difference cannot be explained by hepatic conversion of DEHP to its monoester MEHP, because studies in rats demonstrate that orally administered DEHP undergoes presystemic hydrolysis to MEHP in the gut (Kessler et al. 2004; Pollack et al. 1985). Possible explanations for these results include one or more of the following: *a*) the transactivation assay may not be an accurate quantitative indicator of *in vivo* receptor activation; *b*) the rate and nature of effects downstream of PPAR-α activation may depend on the ligand; or *c*) rate-limiting events independent of PPAR-α activation may contribute to mouse hepatocarcinogenesis by the agonists examined.

### Hepatomegaly, DNA synthesis, and peroxisome proliferation in rats

Table 1 compares potency indicators for various precursor effects at the TD_50_ for four PPAR-α agonists and rat hepatocarcinogens. Our analysis of whether there are consistent levels of *in vivo* precursor effect induction across peroxisome proliferators at the TD_50_ does not include all of the data from a similar, prior analysis by Ashby et al. (1994), for several reasons. First, unlike the CPDB, Ashby et al. did not adjust carcinogenicity data for less-than-lifetime dosing, which is relevant for most compounds. Second, for those mouse carcinogens reported in the CPDB, only acute data are available regarding DNA synthesis effects from Ashby et al. (1994). Therefore, our analysis was restricted to rat precursor and potency data for the four compounds Wy-14,643, nafenopin, clofibrate, and DEHP and included both 1-week and 13-week data to separately address transient and sustained changes in DNA synthesis. Even for this small set of compounds, several limitations in the rat database were apparent. Because no single study provided comparative data for the precursor end points of interest, we used four separate reports. In the Wada et al. (1992) and Tanaka et al. (1992) studies of Wy-14,643 and clofibrate, respectively, administered doses were within 10% of the TD_50_. However, nafenopin data were available only at a single dose of 500 ppm (Lake et al. 1993), which we linearly interpolated to the TD_50_. The highest administered dose of DEHP was 12,500 ppm (David et al. 1999), a dose notably less than the TD_50_, and thus a lower limit based on the assumption of monotonicity with dose is shown. A further data limitation is that in the CPDB, the TD_50_ for only one of the four compounds, DEHP, incorporates data from studies administering more than one dose for 2 years.

The results shown in Table 1 indicate that potency for the occurrence of short-term *in vivo* markers of PPAR-α activation varies widely in magnitude and lacks any apparent correlation with carcinogenic potency. Such differences have been noted previously. Similar to the results presented in Table 1, Marsman et al. (1988) noted that although DEHP (12,000 ppm) and Wy-14,643 (1,000 ppm) induced a similar extent of hepatomegaly and peroxisome proliferation (measured either morphologically or biochemically) after 1 year, the frequency of hepatocelluar lesions was > 100-fold higher in Wy-14,643–exposed relative to DEHP-exposed rats. In addition, a higher labeling index was reported for 12,500 ppm DEHP than the maximal level attained after 50–1,000 ppm Wy-14,643 (David et al. 1999; Tanaka et al. 1992; Wada et al. 1992). We did not examine such differences in maximal responses in our analysis. We also do not present differences in response with dose and time seen among PPAR-α agonists, which are prominent enough to prevent displaying dose–response data on a common scale. For instance, labeling index, which is a measure of DNA synthesis, is increased in a dose-dependent manner at 1 week by clofibrate (1,500, 4,500, and 9,000 ppm) but is decreased compared with controls at 13 weeks at the two higher doses (Tanaka et al. 1992). Together, these findings underscore the significant chemical-specific quantitative differences in these markers that limit their utility for predicting carcinogenic dose–response relationships.

## To What Extent Are Humans Sensitive to PPAR-α Agonists?

Toxicodynamic differences across species, including in the absolute or allometrically scaled amount or activity of the receptor, may contribute to differences in sensitivity of response to PPAR-α agonists. Absolute levels of PPAR-α are generally thought to be lower in human compared with rodent liver. However, PPAR-α amount varies by an order of magnitude among individuals (Palmer et al. 1998; Tugwood et al. 1996); for example, one of the six human samples examined expressed levels comparable to the mouse in one study (Walgren et al. 2000). The pattern of PPAR-α expression across tissues also differs across species (Melnick 2001; Tugwood et al. 1996); for example, human levels are higher in kidney and skeletal muscle than in liver, whereas the highest rodent levels are in liver and kidney. In addition, considerable inter-individual variation in PPAR-α structure and function among humans has been reported (Tugwood et al. 1996), and polymorphisms have been shown to increase or decrease receptor levels and to modulate baseline lipid and apolipoprotein levels, atherosclerotic progression, and the presence of diabetes mellitus and insulin resistance (Flavell et al. 2002, 2005; Foucher et al. 2004; Jamshidi et al. 2002; Tai et al. 2006; Tanaka et al. 2007). An impact of PPAR-α polymorphisms on preexisting disease status and response to PPAR-α agonists is also suggested from bezafibrate [2-(4-{2-[(4-chlorophenyl)formamido]ethyl}phenoxy)-2-methylpropanoic acid] and gemfibrozil [5-(2,5-dimethylphenoxy)-2,2-dimethyl-pentanoic acid] trials (Jamshidi et al. 2002; Tai et al. 2006).

The human PPAR-α is functional in *in vitro* transactivation assays and is responsive to a number of PPAR-α agonists (e.g., nafenopin, clofibrate, and WY-14,643) (Maloney and Waxman 1999; Mukherjee et al. 1994; Sher et al. 1993). Compared with the mouse PPAR-α, human PPAR-α is suggested to be 10- to 20-fold less responsive to Wy-14,643 (Maloney and Waxman 1999; Mukherjee et al. 1994; Palmer et al. 1998). However, this magnitude of interspecies difference has not been demonstrated for other compounds. Hurst and Waxman (2003) reported a 5-fold lower sensitivity to the DEHP metabolite MEHP of human compared with mouse PPAR-α (EC_50_ = 3.2 μM vs. 0.6 μM) in transfected COS-1 monkey kidney cells, but acknowledged that they could not quantify the relative amount of each receptor. Using a similar experimental paradigm, Wolf et al. (2008) found an approximately 2-fold lower slope of the dose–response curve for activation of human compared with mouse PPAR-α for perfluorooctanoic acid and other perfluoroalkyl acids. For other compounds that activate PPAR-α, including TCA and DCA, little (< 2-fold) or no species difference in receptor transactivation sensitivity was evident (Maloney and Waxman 1999). Some compounds appear to more efficiently activate human compared with rodent PPAR-α *in vitro*, as was demonstrated for the synthetic polyunsaturated fatty acid 5,8,11,14-eicosatetraynoic acid in transfected human liver cancer HepG2 cells (Mukherjee et al. 1994) and for perfluorobutane sulfonate in transfected COS-1 monkey kidney cells (Wolf et al. 2008).

Using adenovirus expression in PPAR-α–null mice, Yu et al. (2001) also found little (< 2 fold) or no difference between the mouse and human receptor in terms of induction of *in vivo* markers of peroxisome proliferation. Wy-14,643, ciprofibrate, DEHP, and nafenopin enhanced mRNA and protein levels of peroxisomal genes regardless of whether the human or mouse PPAR-α was expressed (Yu et al. 2001). Transgenic mice stably expressing human PPAR-α in the liver only (Cheung et al. 2004; Morimura et al. 2006) or in all tissues (Yang et al. 2008) of PPAR-α–null mice exhibit increases in both DNA synthesis (with Wy-14,643) and hepatomegaly (with Wy-14,643 and fenofibrate [propan-2-yl 2-{4-[(4-chlorophenyl)carbonyl]phenoxy}-2-methylpropanoate]). However, these increases were much diminished from the response in wild-type mice and lacked statistical significance due largely to the small number of animals studied (*n* = 5–9). With regard to mouse liver tumor induction, Wy-14,643 (1,000 ppm) exposure for up to 44 weeks induced one liver adenoma in 20 PPAR-α-humanized mice, whereas none were seen in 10 untreated animals (Morimura et al. 2006); in comparison, Wy-14,643 (1,000 ppm) caused lethality in 5 of 10 wild-type mice at 38 weeks and tumors in the five surviving animals. These findings are suggestive of differential sensitivity of humanized mice to Wy-14,643. However, the accuracy of estimates of the extent of this difference is limited by the short exposure duration, the substantial mortality and morbidity in wild-type mice, the small number of animals studied, and potential differences in the interaction of the human receptor with mouse-specific coactivators and response elements.

Several key or associative events in the hypothesized MOA have been observed directly in some but not all primate studies (Hoivik et al. 2004; Ito et al. 2007b; Kurata et al. 1998). Studies of cultured primary human hepatocytes have generally reported little or no proliferative response to peroxisome proliferators (for reviews, see Ashby et al. 1994; Peters et al. 2005; Rusyn et al. 2006). The culture conditions, including lack of cocultured nonparenchymal cells (e.g., Kupffer cells), may limit the *in vitro* hepatocyte proliferative response, as observed for other species (e.g., Parzefall et al. 2001). The extent of peroxisome proliferation in human liver after exposure to fibrate drugs (e.g., with clofibrate, gemfibrozil, or fenofibrate) or dialysis treatment (possibly due to DEHP exposure) is reported to be generally less than the rodent response (Blumcke et al. 1983; De La Iglesia et al. 1982; Ganning et al. 1984, 1987; Gariot et al. 1987; Hanefeld et al. 1980, 1983). However, the ability to quantitatively characterize human sensitivity to this effect is limited (e.g., by the small number of subjects studied).

In sum, despite notable qualitative similarities, quantitative differences in receptor activation and the subsequent events in the hypothesized MOA are evident across species. The magnitude of these differences has been best characterized for Wy-14,643, to which rodents appear to have ≥ 10-fold greater sensitivity for response (Cheung et al. 2004; Maloney and Waxman 1999; Morimura et al. 2006; Mukherjee et al. 1994; Palmer et al. 1998; Yu et al. 2001). Although more limited, studies of other compounds that activate PPAR-α suggest a smaller magnitude of difference in sensitivity for response across species than is seen for Wy-14,643 (Hurst and Waxman 2003; Maloney and Waxman 1999; Yu et al. 2001). Considerable interindividual variation in PPAR-α amount, structure, and function has been reported among humans (Tugwood et al. 1996), and some studies have suggested variability in human response to PPAR-α agonists (Jamshidi et al. 2002; Tai et al. 2006). However, few studies have directly examined how these factors may affect sensitivity—as well as the potential for heterogeneity of response—to hepatocarcinogenesis induced by PPAR-α agonists in humans.

## Do Human Epidemiologic Data on Fibrates Offer an Indirect Test of the PPAR-α Activation MOA Hypothesis?

Human exposures to exogenous and endogenous PPAR-α agonists encompass a broad group of chemicals, including environmental contaminants known to activate the receptor, as well as a number of therapeutic agents whose molecular target is one or more receptors in the PPAR family. Indeed, fibrate drugs were developed using rodent models to treat hyperlipidemia in humans before the receptor was identified. These agents have varying degrees of affinity for PPAR-α (Shearer and Hoekstra 2003), and some have multiple mechanisms of action. Drugs that have PPAR-α agonist activity include fibrates or fibric acid derivatives (which are primarily PPAR-α agonists), bezafibrate (which also shows PPAR-γ activity), dual PPAR-α/γ agonists currently under development, the glitazones, and nonsteriod anti-inflammatory drugs (e.g., ibuprofen) (Sertznig et al. 2007).

Some human data on PPAR-α agonist effects are available from fibrate clinical trials and population case–control studies of site-specific cancer (Bezafibrate Infarction Prevention Study Group 1992, 2000; Canner et al. 1986; Committee of Principal Investigators 1978, 1980, 1984; Coronary Drug Research Group 1975, 1977; De Faire et al. 1995; Diabetes Atherosclerosis Intervention Study Investigators 2001; Freeman et al. 2006; Frick et al. 1987, 1997; Huttunen et al. 1994; Keech et al. 2005, 2006; Meade 2001; Rubins et al. 1993, 1999; Tenkanen et al. 2006). These studies examined a range of human responses to PPAR-α agonists, which included atherosclerosis, cardiovascular disease, serum biomarkers of fatty acid metabolism, acute toxicity, and, more limitedly, organ-specific chronic toxicity, including cancer. However, examination of hepatotoxicity in the fibrate clinical trials has been limited to alterations in hepatic metabolic pathways and changes in liver enzymes as assessments of drug tolerance, because the primary focus of these trials was cardiovascular events.

Past reviews of the PPAR-α activation MOA hypothesis have generally focused on liver cancer response in two fibrate clinical trials, the Helsinki Heart Study (Frick et al. 1987; Huttunen et al. 1994; Tenkanen et al. 2006) and the World Health Organization’s Cooperative Trial on Primary Prevention of Ischemic Heart Disease (Committee of Principal Investigators 1978, 1980, 1984), and have concluded that, although limited, those data did not provide evidence of an increased liver cancer risk from fibrate exposure (Ashby et al. 1994; Klaunig et al. 2003). However, the available studies have low power to detect statistical differences in the risk of liver cancer; an estimated five or fewer liver cancer deaths would have been expected in these studies using data from the National Cancer Institute’s Surveillance, Epidemiology, and End Results database (Ries et al. 2008). This low statistical power, in addition to the studies’ exclusion or removal of subjects showing signs of liver (or other) toxicity from treatment, precludes a strong conclusion about the presence or lack of liver cancer risk. These studies and the other fibrate trials did not examine site-specific causes of mortality or morbidity and did not follow subjects for a sufficient period to adequately consider cancer latency; in addition, placebo subjects were offered fibrate therapy at the end of the clinical trials, making analyses after further follow-up difficult to interpret. For example, the three trials that did assess mortality after a follow-up period > 10 years included liver cancers in a larger category of contiguous sites or in the category of all cancers, introducing disease misclassification and a downward bias for any site-specific treatment-related cancers (Canner et al. 1986; Committee of Principal Investigators 1978, 1980, 1984; Huttunen et al. 1994; Tenkanen et al. 2006). In voluntary postmarketing safety reports to the U.S. Food and Drug Administration (FDA), rates of liver adverse event reports for gemfibrozil and fenofibrate (2.6 and 6.9 per 1,000,000 prescriptions, respectively) were similar to those of statins (Holoshitz et al. 2008). However, an examination of liver cancer is precluded by the general underreporting of chronic toxicities to FDA, and the lack of specific FDA reporting requirements for cancer, even premarketing. Because of these inadequacies, the available epidemiologic data for fibrate drugs cannot inform conclusions about the relevance of PPAR-α activation to human cancer.

## Conclusions

As summarized in Figure 1D, the data reviewed and analyses presented in this article raise questions about whether the hypothesized PPAR-α activation MOA is either necessary or sufficient for rodent hepatocarcinogenesis. PPAR-α activation clearly plays a significant role in mouse liver tumor induction by some agonists, such as Wy-14,643. However, the recent study by Ito et al. (2007a) suggests that DEHP can induce PPAR-α–independent tumors without any loss of potency. In addition, as demonstrated by Yang et al. (2007) in their transgenic model of PPAR-α activation in hepatocytes, a robust hepatocyte and peroxisome proliferative response is itself insufficient to cause tumorigenesis. Despite their potential limitations, these studies cast doubt on whether the proposed “key events” such as hepatocyte proliferation play a causal role in tumorigenesis or are merely correlated with cancer, as has been suggested for peroxisome proliferation in prior reviews (e.g., Klaunig et al. 2003). The limited database of other studies that empirically challenge the necessity or sufficiency of the PPAR-α activation MOA in hepatocarcinogenesis per se also motivates a reexamination of whether this MOA hypothesis should be used as the basis for dismissing the human relevance of effects observed in laboratory animals. Of particular concern is whether human health risk assessment MOA conclusions should be based exclusively on evidence of PPAR-α activation and other key events in the hypothesized PPAR-α activation MOA, given that other modes, mechanisms, toxicity pathways and molecular targets may contribute to or be required for the observed adverse effects. Indeed, for most PPAR-α agonists, chemical-specific data to define the range of effects that may contribute to human carcinogenesis are insufficient. Similarly, the epidemiologic data are inadequate to inform conclusions of human relevance.

One possibility that deserves further consideration is that the main determinants of carcinogenicity may not be captured in the hypothesized PPAR-α activation MOA. A recent review (Rusyn et al. 2006) addressed other mechanistic effects of DEHP and proposed that tumors arise from a combination of molecular signals and pathways, rather than from a single event such as PPAR-α activation. Indeed, the compounds that activate PPAR-α are pleiotropic and have been reported to exhibit a diversity of responses in addition to the hallmark effect of peroxisome proliferation, including genotoxicity (reviewed by Melnick 2001), epigenetic alterations (e.g., hypomethylation) (Pogribny et al. 2007), oxidative stress (reviewed in O’Brien et al. 2005), and effects on other receptors (e.g., Guo et al. 2007) and other organelles (e.g., mitochondria) within parenchymal cells (Lundgren et al. 1987; Scatena et al. 2003; Youssef and Badr 1998; Zhou and Wallace 1999). Importantly, several of these compounds (e.g., Wy-14,643, nafenopin, and DEHP) reportedly affect nonparenchymal liver cells that do not express PPAR-α (Parzefall et al. 2001; Peters et al. 2000; Rose et al. 1997, 1999; Rusyn et al. 2001) as well as other organ systems. Not all precursor, toxic, or carcinogenic effects rely solely on PPAR-α; for example, in PPAR-α–null mice, DEHP causes adverse testicular and renal effects at 24 weeks (Ward et al. 1998) as well as hepatocarcinogenicity at 2 years (Ito et al. 2007a). Indeed, with regard to the hepatic, testicular, and pancreatic cancers associated with phthalate exposures, a report of the National Research Council (2008b) concluded that “there is evidence that these cancer types may be mediated by mechanisms independent of PPARα.” Therefore, the adequacy of the scientific basis for the conclusion that PPAR-α agonists pose no carcinogenic risk to humans requires reexamination.

## Perspectives and Future Directions

Our exploration of the hypothesized PPAR-α activation MOA highlights some of the critical challenges pertinent to the evaluation of mechanistic data and its use in decisions regarding the relative human sensitivity for biological effects observed in laboratory animals. Primary among these are differentiating causative from associative events in a proposed MOA, addressing multiple MOA hypotheses, and considering how Hill’s considerations have been adopted to address these key questions. Alternatives to the use of Hill’s considerations for assessing causality for toxicologic data have been discussed (Gray et al. 2001; Guzelian et al. 2005; Weed 2005). In addition, alternate conceptualizations of causal inference that allow for consideration of multiple causative factors may be instructive for MOA data evaluation. In particular, the sufficient-component cause model described originally by Rothman (1976) may provide useful theoretical and conceptual bases pertinent to evaluating MOA data and generating new MOA hypotheses. This model facilitates the conceptualization of hypotheses regarding multiple causes contributing to a single disease of interest and addresses such issues as multiple diseases arising from a single cause and multiple causes for a single disease with distinct models of susceptibility and biologic interactions, as well as background risk due to endogenous processes or other exposures. These “casual inference” methodologies may be useful in identifying the candidate key events, MOAs, or toxicity pathways that individually or together constitute the operant MOAs. Thereafter, direct experimentation is needed, and thus a critical aspect of MOA data evaluation is to consider the extent to which hypotheses have been challenged experimentally, according to the scientific method.

With regard to considerations of human sensitivity to any given MOA, our analyses support a change in focus from using quantitative data to make a “binary” determination of “relevant”/“not relevant” during hazard identification to a data-driven approach of using available quantitative data during dose–response estimation. This includes incorporating data-derived estimates of the degree of inter- as well as intraspecies differences in sensitivity to identified causal events in a hypothesized MOA during risk evaluation. The extent to which such quantitative approaches rely on a single MOA should be commensurate with the experimental support that the hypothesized MOA is the sole causative factor for carcinogenesis. These issues are especially important if the hypothesized MOA would be used as the basis for dismissing the human relevance of effects observed in laboratory animal models.

These considerations also highlight the need for a more robust database for compounds of environmental concern that activate PPAR-α, such as phthalates, perfluorinated acids, chlorinated solvents, and chloroacetic acids, either alone or in combinations relevant to human exposures. The extensive research focus on Wy-14,643 is particularly problematic, because *a*) it is typically administered at necrogenic doses well above those required for maximal responses; *b*) it is one of the few agonists that produce sustained, as opposed to only transient, enhancement of DNA synthesis in hepatocytes; *c*) unlike many other agonists, it preferentially activates rodent forms of PPAR-α (with humans exhibiting ~20-fold less sensitivity); and *d*) humans apparently have never been exposed to it, either in an experimental or clinical setting. Although it is assumed to be a prototype for the class, these considerations limit the general applicability of the research findings with Wy-14,643 to the diverse chemicals that activate PPAR-α. Rather than using Wy-14,643 and focusing exclusively on correlative effects in the hypothesized PPAR-α activation MOA in hepatocytes, experimental studies are needed that aim to elucidate and characterize the pleiotropic effects of PPAR-α agonists. Such research could broadly explore other mechanisms and identify the toxicity pathways responsible for effects that are known to be associated with human carcinogenesis. Based on this research, plausible MOA hypotheses for hepatocarcinogenesis induced by these environmental contaminants could be generated and ultimately tested using the scientific method.

## Figures and Tables

**Table 1:** Potency indicators for rat hepatocarcinogenicity and common short-term markers of PPAR-α activation for four PPAR-α agonists.

Chemical	Tumor TD_50_ (ppm in diet)	Fold increase over control at tumor TD_50_					
		1 week			13 weeks		
		RLW	LI	PCO	RLW	LI	PCO
Wy-14,643	109	1.8	12.0	13.0	2.6	6.8	39.0
Nafenopin	275	1.4	3.6	7.6	1.5	1.12	6.7
Clofibrate	4,225	1.4	4.4	4.2	1.4	0.95	3.7
DEHP	17,900	≥1.4	≥ 19.0	≥ 3.6	≥ 1.9	≥ 1.25	≥ 4.9

Abbreviations: LI, labeling index; PCO, cyanide-insensitive palmitoyl CoA oxidation; RLW, relative liver weight; TD_50_, daily dose inducing tumors in half of the mice that would otherwise have remained tumor-free. For ease of comparison with precursor effect studies, administered doses for the tumor TD_50_ values in the CPDB were back-converted to equivalent parts per million in diet using the formula of Gold et al. (2005): TD_50_ (mg/kg-day) = TD_50_ (ppm in diet) × 0.04 (for male rats). Administered doses for precursor data on Wy-14,643 (Wada et al. 1992) and clofibrate (Tanaka et al. 1992) were within 10% of the TD_50_. Because nafenopin precursor data were available only at 0 and 500 ppm (Lake et al. 1993), these doses were linearly interpolated to the TD_50_. Because the highest administered dose of DEHP in precursor effect studies was 12,500 ppm (David et al. 1999), a lower limit is shown, based on the assumption of monotonicity with dose.

**Table 2:** Potency indicators for mouse hepatocarcinogenicity and *in vitro* transactivation of mouse PPAR-α for four PPAR-α agonists.

Hepatocarcinogen	Carcinogenic potency indicator (TD_50_; mg/kg-day)	Transactivation potency indicators (μM)	
		EC_50_	EC_2-fold_
Wy-14,643	< 10.8	0.63	~0.4
DCA	119.0	~800	~600
TCA	584.0	~500	~300
DEHP/MEHP	700.0	~0.7	~0.7

The “<“ symbol denotes an upper limit due to maximal response. A “~” symbol indicates that the transactivation potency was approximated (to one significant figure) from figures or tables in Maloney and Waxman (1999).

## References

[b1-ehp-117-1664] Ashby J, Brady A, Elcombe CR, Elliott BM, Ishmael J, Odum J (1994). Mechanistically-based human hazard assessment of peroxisome proliferator-induced hepatocarcinogenesis. Hum Exp Toxicol.

[b2-ehp-117-1664] Barrass NC, Price RJ, Lake BG, Orton TC (1993). Comparison of the acute and chronic mitogenic effects of the peroxisome proliferators methylclofenapate and clofibric acid in rat liver. Carcinogenesis.

[b3-ehp-117-1664] Bezafibrate Infarction Prevention Study Group (1992). Lipids and lipoproteins in symptomatic coronary heart disease. Distribution, intercorrelations, and significance for risk classification in 6,700 men and 1,500 women. Circulation.

[b4-ehp-117-1664] Bezafibrate Infarction Prevention Study Group (2000). Secondary prevention by raising HDL cholesterol and reducing triglycerides in patients with coronary artery disease: the Bezafibrate Infarction Prevention (BIP) study. Circulation.

[b5-ehp-117-1664] Blumcke S, Schwartzkopff W, Lobeck H, Edmondson NA, Prentice DE, Blane GF (1983). Influence of fenofibrate on cellular and subcellular liver structure in hyperlipidemic patients. Atherosclerosis.

[b6-ehp-117-1664] Caldwell JC, Keshava N, Evans MV (2008). Difficulty of mode of action determination for trichloroethylene: an example of complex interactions of metabolites and other chemical exposures. Environ Mol Mutagen.

[b7-ehp-117-1664] Canner PL, Berge KG, Wenger NK, Stamler J, Friedman L, Prineas RJ (1986). Fifteen year mortality in Coronary Drug Project patients: long-term benefit with niacin. J Am Coll Cardiol.

[b8-ehp-117-1664] Cheung C, Akiyama TE, Ward JM, Nicol CJ, Feigenbaum L, Vinson C (2004). Diminished hepatocellular proliferation in mice humanized for the nuclear receptor peroxisome proliferator-activated receptor alpha. Cancer Res.

[b9-ehp-117-1664] Committee of Principal Investigators (1978). A cooperative trial in the primary prevention of ischaemic heart disease using clofibrate. Report from the Committee of Principal Investigators. Br Heart J.

[b10-ehp-117-1664] Committee of Principal Investigators (1980). W.H.O. cooperative trial on primary prevention of ischaemic heart disease using clofibrate to lower serum cholesterol: mortality follow-up. Report of the Committee of Principal Investigators. Lancet.

[b11-ehp-117-1664] Committee of Principal Investigators (1984). WHO cooperative trial on primary prevention of ischaemic heart disease with clofibrate to lower serum cholesterol: final mortality follow-up. Report of the Committee of Principal Investigators. Lancet.

[b12-ehp-117-1664] Conway JG, Tomaszewski KE, Olson MJ, Cattley RC, Marsman DS, Popp JA (1989). Relationship of oxidative damage to the hepatocarcinogenicity of the peroxisome proliferators di(2-ethylhexyl)phthalate and Wy-14,643. Carcinogenesis.

[b13-ehp-117-1664] Coronary Drug Research Group (1975). Clofibrate and niacin in coronary heart disease. JAMA.

[b14-ehp-117-1664] Coronary Drug Research Group (1977). Gallbladder disease as a side effect of drugs influencing lipid metabolism. Experience in the Coronary Drug Project. N Engl J Med.

[b15-ehp-117-1664] David RM, Moore MR, Cifone MA, Finney DC, Guest D (1999). Chronic peroxisome proliferation and hepatomegaly associated with the hepatocellular tumorigenesis of di(2-ethylhexyl)phthalate and the effects of recovery. Toxicol Sci.

[b16-ehp-117-1664] De Faire U, Ericsson C-G, Hamsten A, Nilsson J, for the Bezafibrate Coronary Atherosclerosis Intervention Trial Investigators (1995). Design features of a five-year Bezafibrate Coronary Atherosclerosis Intervention Trial. Drugs Exp Clin Res.

[b17-ehp-117-1664] De La Iglesia FA, Lewis JE, Buchanan RA, Marcus EL, McMahon G (1982). Light and electron microscopy of liver in hyperlipoproteinemic patients under long-term gemfibrozil treatment. Atherosclerosis.

[b18-ehp-117-1664] Diabetes Atherosclerosis Intervention Study Investigators (2001). Effect of fenofibrate on progression of coronary-artery disease in type 2 diabetes: the Diabetes Atherosclerosis Intervention Study, a randomised study. Lancet.

[b19-ehp-117-1664] Eacho PI, Lanier TL, Brodhecker CA (1991). Hepatocellular DNA synthesis in rats given peroxisome proliferating agents: comparison of WY-14,643 to clofibric acid, nafenopin and LY171883. Carcinogenesis.

[b20-ehp-117-1664] Flavell DM, Ireland H, Stephens JW, Hawe E, Acharya J, Mather H (2005). Peroxisome proliferator-activated receptor alpha gene variation influences age of onset and progression of type 2 diabetes. Diabetes.

[b21-ehp-117-1664] Flavell DM, Jamshidi Y, Hawe E, Pineda Torra I, Taskinen MR, Frick MH (2002). Peroxisome proliferator-activated receptor alpha gene variants influence progression of coronary atherosclerosis and risk of coronary artery disease. Circulation.

[b22-ehp-117-1664] Foucher C, Rattier S, Flavell DM, Talmud PJ, Humphries SE, Kastelein JJ (2004). Response to micronized fenofibrate treatment is associated with the peroxisome-proliferator-activated receptors alpha G/C intron7 polymorphism in subjects with type 2 diabetes. Pharmacogenetics.

[b23-ehp-117-1664] Freeman SR, Drake AL, Heilig LF, Graber M, McNealy K, Schilling LM (2006). Statins, fibrates, and melanoma risk: a systematic review and meta-analysis. J Natl Cancer Inst.

[b24-ehp-117-1664] Frick MH, Elo O, Haapa K, Heinonen OP, Heinsalmi P, Helo P (1987). Helsinki Heart Study: primary-prevention trial with gemfibrozil in middle-aged men with dyslipidemia. Safety of treatment, changes in risk factors, and incidence of coronary heart disease. N Engl J Med.

[b25-ehp-117-1664] Frick MH, Syvanne M, Nieminen MS, Kauma H, Majahalme S, Virtanen V (1997). Prevention of the angiographic progression of coronary and vein-graft atherosclerosis by gemfibrozil after coronary bypass surgery in men with low levels of HDL cholesterol. Lopid Coronary Angiography Trial (LOCAT) Study Group. Circulation.

[b26-ehp-117-1664] Ganning AE, Brunk U, Dallner G (1984). Phthalate esters and their effect on the liver. Hepatology.

[b27-ehp-117-1664] Ganning AE, Brunk U, Edlund C, Elhammer A, Dallner G (1987). Effects of prolonged administration of phthalate ester on the liver. Environ Health Perspect.

[b28-ehp-117-1664] Gariot P, Barrat E, Drouin P, Genton P, Pointel JP, Foliguet B (1987). Morphometric study of human hepatic cell modifications induced by fenofibrate. Metabolism.

[b29-ehp-117-1664] Gold LS, Manley NB, Slone TH, Rohrbach L, Garfinkel GB (2005). Supplement to the Carcinogenic Potency Database (CPDB): results of animal bioassays published in the general literature through 1997 and by the National Toxicology Program in 1997–1998. Toxicol Sci.

[b30-ehp-117-1664] Gray GM, Baskin SI, Charnley G, Cohen JT, Gold LS, Kekvliet NI (2001). The Annapolis Accords on the use of toxicology in risk assessment and decision-making: an Annapolis Center workshop report. Toxicol Methods.

[b31-ehp-117-1664] Gray TJ, Beamand JA, Lake BG, Foster JR, Gangolli SD (1982). Peroxisome proliferation in cultured rat hepatocytes produced by clofibrate and phthalate ester metabolites. Toxicol Lett.

[b32-ehp-117-1664] Gray TJ, Lake BG, Beamand JA, Foster JR, Gangolli SD (1983). Peroxisome proliferation in primary cultures of rat hepatocytes. Toxicol Appl Pharmacol.

[b33-ehp-117-1664] Guo D, Sarkar J, Suino-Powell K, Xu Y, Matsumoto K, Jia Y (2007). Induction of nuclear translocation of constitutive androstane receptor by peroxisome proliferator-activated receptor alpha synthetic ligands in mouse liver. J Biol Chem.

[b34-ehp-117-1664] Guyton KZ, Barone S, Brown RC, Euling SY, Jinot J, Makris S (2008). Mode of action frameworks: a critical analysis. J Toxicol Environ Health B Crit Rev.

[b35-ehp-117-1664] Guzelian PS, Victoroff MS, Halmes NC, James RC, Guzelian CP (2005). Evidence-based toxicology: a comprehensive framework for causation. Hum Exp Toxicol.

[b36-ehp-117-1664] Hanefeld M, Kemmer C, Kadner E (1983). Relationship between morphological changes and lipid-lowering action of p-chlor-phenoxyisobutyric acid (CPIB) on hepatic mitochondria and peroxisomes in man. Atherosclerosis.

[b37-ehp-117-1664] Hanefeld M, Kemmer C, Leonhardt W, Kunze KD, Jaross W, Haller H (1980). Effects of p-chlorophenoxyisobutyric acid (CPIB) on the human liver. Atherosclerosis.

[b38-ehp-117-1664] Hasmall SC, James NH, Macdonald N, West D, Chevalier S, Cosulich SC (1999). Suppression of apoptosis and induction of DNA synthesis in vitro by the phthalate plasticizers monoethylhexylphthalate (MEHP) and diisononylphthalate (DINP): a comparison of rat and human hepatocytes in vitro. Arch Toxicol.

[b39-ehp-117-1664] Hill AB (1965). The environment and disease: association or causation?. Proc R Soc Med.

[b40-ehp-117-1664] Hoivik DJ, Qualls CW, Mirabile RC, Cariello NF, Kimbrough CL, Colton HM (2004). Fibrates induce hepatic peroxisome and mitochondrial proliferation without overt evidence of cellular proliferation and oxidative stress in cynomolgus monkeys. Carcinogenesis.

[b41-ehp-117-1664] Holoshitz N, Alsheikh-Ali AA, Karas RH (2008). Relative safety of gemfibrozil and fenofibrate in the absence of concomitant cerivastatin use. Am J Cardiol.

[b42-ehp-117-1664] Howroyd P, Swanson C, Dunn C, Cattley RC, Corton JC (2004). Decreased longevity and enhancement of age-dependent lesions in mice lacking the nuclear receptor peroxisome proliferator-activated receptor alpha (PPARalpha). Toxicol Pathol.

[b43-ehp-117-1664] Hurst CH, Waxman DJ (2003). Activation of PPARalpha and PPARgamma by environmental phthalate monoesters. Toxicol Sci.

[b44-ehp-117-1664] Huttunen JK, Heinonen OP, Manninen V, Koskinen P, Hakulinen T, Teppo L (1994). The Helsinki Heart Study: an 8.5-year safety and mortality follow-up. J Intern Med.

[b45-ehp-117-1664] IARC (2000). Di(2-ethylhexyl) phthalate. IARC Monogr Eval Carcinog Risk Chem Hum.

[b46-ehp-117-1664] Issemann I, Green S (1990). Activation of a member of the steroid hormone receptor superfamily by peroxisome proliferators. Nature.

[b47-ehp-117-1664] Ito Y, Yamanoshita O, Asaeda N, Tagawa Y, Lee CH, Aoyama T (2007a). Di(2-ethylhexyl)phthalate induces hepatic tumorigenesis through a peroxisome proliferator-activated receptor alpha-independent pathway. J Occup Health.

[b48-ehp-117-1664] Ito Y, Yamanoshita O, Kurata Y, Kamijima M, Aoyama T, Nakajima T (2007b). Induction of peroxisome proliferator-activated receptor alpha (PPARalpha)-related enzymes by di(2-ethylhexyl) phthalate (DEHP) treatment in mice and rats, but not marmosets. Arch Toxicol.

[b49-ehp-117-1664] Ito Y, Yokota H, Wang R, Yamanoshita O, Ichihara G, Wang H (2005). Species differences in the metabolism of di(2-ethylhexyl) phthalate (DEHP) in several organs of mice, rats, and marmosets. Arch Toxicol.

[b50-ehp-117-1664] Jamshidi Y, Flavell DM, Hawe E, MacCallum PK, Meade TW, Humphries SE (2002). Genetic determinants of the response to bezafibrate treatment in the lower extremity arterial disease event reduction (LEADER) trial. Atherosclerosis.

[b51-ehp-117-1664] Keech A, Simes RJ, Barter P, Best J, Scott R, Taskinen MR (2006). Correction to the FIELD study report. Lancet.

[b52-ehp-117-1664] Keech A, Simes RJ, Barter P, Best J, Scott R, Taskinen MR (2005). Effects of long-term fenofibrate therapy on cardiovascular events in 9795 people with type 2 diabetes mellitus (the FIELD study): randomised controlled trial. Lancet.

[b53-ehp-117-1664] Kessler W, Numtip W, Grote K, Csanády GA, Chahoud I, Filser JG (2004). Blood burden of di(2-ethylhexyl) phthalate and its primary metabolite mono(2-ethylhexyl) phthalate in pregnant and nonpregnant rats and marmosets. Toxicol Appl Pharmacol.

[b54-ehp-117-1664] Klaunig JE, Babich MA, Baetcke KP, Cook JC, Corton JC, David RM (2003). PPARalpha agonist-induced rodent tumors: modes of action and human relevance. Crit Rev Toxicol.

[b55-ehp-117-1664] Kurata Y, Kidachi F, Yokoyama M, Toyota N, Tsuchitani M, Katoh M (1998). Subchronic toxicity of Di(2-ethylhexyl) phthalate in common marmosets: lack of hepatic peroxisome proliferation, testicular atrophy, or pancreatic acinar cell hyperplasia. Toxicol Sci.

[b56-ehp-117-1664] Lake BG, Evans JG, Cunninghame ME, Price RJ (1993). Comparison of the hepatic effects of nafenopin and WY-14,643 on peroxisome proliferation and cell replication in the rat and Syrian hamster. Environ Health Perspect.

[b57-ehp-117-1664] Lee SS, Pineau T, Drago J, Lee EJ, Owens JW, Kroetz DL (1995). Targeted disruption of the alpha isoform of the peroxisome proliferator-activated receptor gene in mice results in abolishment of the pleiotropic effects of peroxisome proliferators. Mol Cell Biol.

[b58-ehp-117-1664] Lundgren B, Meijer J, DePierre JW (1987). Induction of cytosolic and microsomal epoxide hydrolases and proliferation of peroxisomes and mitochondria in mouse liver after dietary exposure to p-chlorophenoxyacetic acid, 2,4-dichlorophenoxyacetic acid and 2,4,5-trichlorophenoxyacetic acid. Biochem Pharmacol.

[b59-ehp-117-1664] Maloney EK, Waxman DJ (1999). trans-Activation of PPARalpha and PPARgamma by structurally diverse environmental chemicals. Toxicol Appl Pharmacol.

[b60-ehp-117-1664] Marsman DS, Cattley RC, Conway JG, Popp JA (1988). Relationship of hepatic peroxisome proliferation and replicative DNA synthesis to the hepatocarcinogenicity of the peroxisome proliferators di(2-ethylhexyl)phthalate and [4-chloro-6-(2,3-xylidino)-2-pyrimidinylthio]acetic acid (Wy-14,643) in rats. Cancer Res.

[b61-ehp-117-1664] Marsman DS, Goldsworthy TL, Popp JA (1992). Contrasting hepatocytic peroxisome proliferation, lipofuscin accumulation and cell turnover for the hepatocarcinogens Wy-14,643 and clofibric acid. Carcinogenesis.

[b62-ehp-117-1664] Meade TW (2001). Design and intermediate results of the Lower Extremity Arterial Disease Event Reduction (LEADER)* trial of bezafibrate in men with lower extremity arterial disease [ISRCTN4119421]. Curr Control Trials Cardiovasc Med.

[b63-ehp-117-1664] Melnick RL (2001). Is peroxisome proliferation an obligatory precursor step in the carcinogenicity of di(2-ethylhexyl) phthalate (DEHP)?. Environ Health Perspect.

[b64-ehp-117-1664] Melnick RL, Huff J, Barrett JC, Maronpot RR, Lucier G, Portier CJ (1993). Cell proliferation and chemical carcinogenesis: symposium overview. Environ Health Perspect.

[b65-ehp-117-1664] Mitchell AM, Bridges JW, Elcombe CR (1984). Factors influencing peroxisome proliferation in cultured rat hepatocytes. Arch Toxicol.

[b66-ehp-117-1664] Mitchell FE, Price SC, Hinton RH, Grasso P, Bridges JW (1985). Time and dose-response study of the effects on rats of the plasticizer di(2-ethylhexyl) phthalate. Toxicol Appl Pharmacol.

[b67-ehp-117-1664] Morimura K, Cheung C, Ward JM, Reddy JK, Gonzalez FJ (2006). Differential susceptibility of mice humanized for peroxisome proliferator-activated receptor alpha to Wy-14,643-induced liver tumorigenesis. Carcinogenesis.

[b68-ehp-117-1664] Mukherjee R, Jow L, Noonan D, McDonnell DP (1994). Human and rat peroxisome proliferator activated receptors (PPARs) demonstrate similar tissue distribution but different responsiveness to PPAR activators. J Steroid Biochem Mol Biol.

[b69-ehp-117-1664] National Research Council (2008a). Science and Decisions: Advancing Risk Assessment.

[b70-ehp-117-1664] National Research Council (2008b). Phthalates and Cumulative Risk Assessment: The Task Ahead.

[b71-ehp-117-1664] O’Brien ML, Spear BT, Glauert HP (2005). Role of oxidative stress in peroxisome proliferator-mediated carcinogenesis. Crit Rev Toxicol.

[b72-ehp-117-1664] Palmer CN, Hsu MH, Griffin KJ, Raucy JL, Johnson EF (1998). Peroxisome proliferator activated receptor-alpha expression in human liver. Mol Pharmacol.

[b73-ehp-117-1664] Parzefall W, Berger W, Kainzbauer E, Teufelhofer O, Schulte-Hermann R, Thurman RG (2001). Peroxisome proliferators do not increase DNA synthesis in purified rat hepatocytes. Carcinogenesis.

[b74-ehp-117-1664] Peters JM, Cattley RC, Gonzalez FJ (1997). Role of PPAR alpha in the mechanism of action of the nongenotoxic carcinogen and peroxisome proliferator Wy-14,643. Carcinogenesis.

[b75-ehp-117-1664] Peters JM, Cheung C, Gonzalez FJ (2005). Peroxisome proliferator-activated receptor-alpha and liver cancer: where do we stand?. J Mol Med.

[b76-ehp-117-1664] Peters JM, Rusyn I, Rose ML, Gonzalez FJ, Thurman RG (2000). Peroxisome proliferator-activated receptor alpha is restricted to hepatic parenchymal cells, not Kupffer cells: implications for the mechanism of action of peroxisome proliferators in hepatocarcinogenesis. Carcinogenesis.

[b77-ehp-117-1664] Pogribny IP, Tryndyak VP, Woods CG, Witt SE, Rusyn I (2007). Epigenetic effects of the continuous exposure to peroxisome proliferator WY-14,643 in mouse liver are dependent upon peroxisome proliferator activated receptor alpha. Mutat Res.

[b78-ehp-117-1664] Pollack GM, Li RC, Ermer JC, Shen DD (1985). Effects of route of administration and repetitive dosing on the disposition kinetics of di(2-ethylhexyl) phthalate and its monode-esterified metabolite in rats. Toxicol Appl Pharmacol.

[b79-ehp-117-1664] Reddy JK, Reddy MK, Usman MI, Lalwani ND, Rao MS (1986). Comparison of hepatic peroxisome proliferative effect and its implication for hepatocarcinogenicity of phthalate esters, di(2-ethylhexyl) phthalate, and di(2-ethylhexyl) adi-pate with a hypolipidemic drug. Environ Health Perspect.

[b80-ehp-117-1664] Ries LAG, Melbert D, Krapcho M, Stinchcomb DG, Howlader N, Horner MJ (2008). SEER Cancer Statistics Review, 1975–2005, Based on November 2007 SEER Data Submission, Posted 2008.

[b81-ehp-117-1664] Rose ML, Germolec DR, Schoonhoven R, Thurman RG (1997). Kupffer cells are causally responsible for the mitogenic effect of peroxisome proliferators. Carcinogenesis.

[b82-ehp-117-1664] Rose ML, Rivera CA, Bradford BU, Graves LM, Cattley RC, Schoonhoven R (1999). Kupffer cell oxidant production is central to the mechanism of peroxisome proliferators. Carcinogenesis.

[b83-ehp-117-1664] Rothman KJ (1976). Causes. Am J Epidemiol.

[b84-ehp-117-1664] Rubins HB, Robins SJ, Collins D, Fye CL, Anderson JW, Elam MB (1999). Gemfibrozil for the secondary prevention of coronary heart disease in men with low levels of high-density lipoprotein cholesterol. Veterans Affairs High-Density Lipoprotein Cholesterol Intervention Trial Study Group. N Engl J Med.

[b85-ehp-117-1664] Rubins HB, Robins SJ, Iwane MK, Boden WE, Elam MB, Fye CL (1993). Rationale and design of the Department of Veterans Affairs High-Density Lipoprotein Cholesterol Intervention Trial (HIT) for secondary prevention of coronary artery disease in men with low high-density lipoprotein cholesterol and desirable low-density lipoprotein cholesterol. Am J Cardiol.

[b86-ehp-117-1664] Rusyn I, Kadiiska MB, Dikalova A, Kono H, Yin M, Tsuchiya K (2001). Phthalates rapidly increase production of reactive oxygen species in vivo: role of Kupffer cells. Mol Pharmacol.

[b87-ehp-117-1664] Rusyn I, Peters JM, Cunningham ML (2006). Modes of action and species-specific effects of di-(2-ethylhexyl)phthalate in the liver. Crit Rev Toxicol.

[b88-ehp-117-1664] Scatena R, Bottoni P, Vincenzoni F, Messana I, Martorana GE, Nocca G (2003). Bezafibrate induces a mitochondrial derangement in human cell lines: a PPAR-independent mechanism for a peroxisome proliferator. Chem Res Toxicol.

[b89-ehp-117-1664] Sertznig P, Seifert M, Tilgen W, Reichrath J (2007). Present concepts and future outlook: function of peroxisome proliferator-activated receptors (PPARs) for pathogenesis, progression, and therapy of cancer. J Cell Physiol.

[b90-ehp-117-1664] Shearer BG, Hoekstra WJ (2003). Recent advances in peroxisome proliferator-activated receptor science. Curr Med Chem.

[b91-ehp-117-1664] Sher T, Yi HF, McBride OW, Gonzalez FJ (1993). cDNA cloning, chromosomal mapping, and functional characterization of the human peroxisome proliferator activated receptor. Biochemistry.

[b92-ehp-117-1664] Tai ES, Collins D, Robins SJ, O’Connor JJ, Bloomfield HE, Ordovas JM (2006). The L162V polymorphism at the peroxisome proliferator activated receptor alpha locus modulates the risk of cardiovascular events associated with insulin resistance and diabetes mellitus: the Veterans Affairs HDL Intervention Trial (VA-HIT). Atherosclerosis.

[b93-ehp-117-1664] Takashima K, Ito Y, Gonzalez FJ, Nakajima T (2008). Different mechanisms of DEHP-induced hepatocellular adenoma tumorigenesis in wild-type and PPAR alpha-null mice. J Occup Health.

[b94-ehp-117-1664] Tanaka K, Smith PF, Stromberg PC, Eydelloth RS, Herold EG, Grossman SJ (1992). Studies of early hepatocellular proliferation and peroxisomal proliferation in Sprague-Dawley rats treated with tumorigenic doses of clofibrate. Toxicol Appl Pharmacol.

[b95-ehp-117-1664] Tanaka T, Ordovas JM, Delgado-Lista J, Perez-Jimenez F, Marin C, Perez-Martinez P (2007). Peroxisome proliferator-activated receptor alpha polymorphisms and postprandial lipemia in healthy men. J Lipid Res.

[b96-ehp-117-1664] Tenkanen L, Manttari M, Kovanen PT, Virkkunen H, Manninen V (2006). Gemfibrozil in the treatment of dyslipidemia: an 18-year mortality follow-up of the Helsinki Heart Study. Arch Intern Med.

[b97-ehp-117-1664] Tugwood JD, Aldridge TC, Lambe KG, Macdonald N, Woodyatt NJ (1996). Peroxisome proliferator-activated receptors: structures and function. Ann N Y Acad Sci.

[b98-ehp-117-1664] U.S. EPA (2005). Guidelines for carcinogen risk assessment and supplemental guidance for assessing susceptibility from early-life exposure to carcinogens. Fed Reg.

[b99-ehp-117-1664] Wada N, Marsman DS, Popp JA (1992). Dose-related effects of the hepatocarcinogen, Wy-14,643, on peroxisomes and cell replication. Fundam Appl Toxicol.

[b100-ehp-117-1664] Walgren JE, Kurtz DT, McMillan JM (2000). Expression of PPAR(alpha) in human hepatocytes and activation by trichloroacetate and dichloroacetate. Res Commun Mol Pathol Pharmacol.

[b101-ehp-117-1664] Walgren JL, Kurtz DT, McMillan JM (2005). Lack of direct mitogenic activity of dichloroacetate and trichloroacetate in cultured rat hepatocytes. Toxicology.

[b102-ehp-117-1664] Ward JM, Peters JM, Perella CM, Gonzalez FJ (1998). Receptor and nonreceptor-mediated organ-specific toxicity of di(2-ethylhexyl)phthalate (DEHP) in peroxisome proliferator-activated receptor alpha-null mice. Toxicol Pathol.

[b103-ehp-117-1664] Weed DL (2005). Weight of evidence: a review of concept and methods. Risk Anal.

[b104-ehp-117-1664] Wolf CJ, Takacs ML, Schmid JE, Lau C, Abbott BD (2008). Activation of mouse and human peroxisome proliferator-activated receptor alpha by perfluoroalkyl acids of different functional groups and chain lengths. Toxicol Sci.

[b105-ehp-117-1664] Woods CG, Heuvel JP, Rusyn I (2007). Genomic profiling in nuclear receptor-mediated toxicity. Toxicol Pathol.

[b106-ehp-117-1664] Yang Q, Ito S, Gonzalez FJ (2007). Hepatocyte-restricted constitutive activation of PPAR alpha induces hepatoproliferation but not hepatocarcinogenesis. Carcinogenesis.

[b107-ehp-117-1664] Yang Q, Nagano T, Shah Y, Cheung C, Ito S, Gonzalez FJ (2008). The PPAR alpha-humanized mouse: a model to investigate species differences in liver toxicity mediated by PPAR alpha. Toxicol Sci.

[b108-ehp-117-1664] Yeldandi AV, Milano M, Subbarao V, Reddy JK, Rao MS (1989). Evaluation of liver cell proliferation during ciprofibrate-induced hepatocarcinogenesis. Cancer Lett.

[b109-ehp-117-1664] Youssef J, Badr M (1998). Extraperoxisomal targets of peroxisome proliferators: mitochondrial, microsomal, and cytosolic effects. Implications for health and disease. Crit Rev Toxicol.

[b110-ehp-117-1664] Yu S, Cao WQ, Kashireddy P, Meyer K, Jia Y, Hughes DE (2001). Human peroxisome proliferator-activated receptor alpha (PPAR alpha) supports the induction of peroxisome proliferation in PPARalpha-deficient mouse liver. J Biol Chem.

[b111-ehp-117-1664] Zhou S, Wallace KB (1999). The effect of peroxisome proliferators on mitochondrial bioenergetics. Toxicol Sci.

